# Stromal POSTN Enhances Motility of Both Cancer and Stromal Cells and Predicts Poor Survival in Colorectal Cancer

**DOI:** 10.3390/cancers15030606

**Published:** 2023-01-18

**Authors:** Akane Ueki, Masayuki Komura, Akira Koshino, Chengbo Wang, Kazuhiro Nagao, Mai Homochi, Yuki Tsukada, Masahide Ebi, Naotaka Ogasawara, Toyonori Tsuzuki, Kenji Kasai, Kunio Kasugai, Satoru Takahashi, Shingo Inaguma

**Affiliations:** 1 Department of Experimental Pathology and Tumor Biology, Nagoya City University Graduate School of Medical Sciences, Nagoya 467-8601, Japan; 2 Division of Gastroenterology, Department of Internal Medicine, Aichi Medical University School of Medicine, Nagakute 408-1195, Japan; 3 Surgical Pathology, Aichi Medical University School of Medicine, Nagakute 408-1195, Japan; 4 Department of Pathology, Aichi Medical University School of Medicine, Nagakute 408-1195, Japan; 5 Department of Pathology, Nagoya City University East Medical Center, Nagoya 464-8547, Japan

**Keywords:** colorectal cancer (CRC), immunohistochemistry, periostin (POSTN), cancer-associated fibroblasts (CAFs), migration

## Abstract

**Simple Summary:**

Evidence for the tumor-supporting capacities of cancer-associated fibroblasts (CAFs) has rapidly been accumulating. The present study revealed that patients with stromal periostin (POSTN)-positive colorectal cancer (CRC) with peritoneal and distant organ metastasis had a significantly worse 5-year survival rate. Furthermore, significant associations between stromal POSTN and complete loss of p53 were identified. POSTN was also associated with decorin and fibroblast-activation protein expression, indicating gene expression status and/or phenotypes similar to CAF-A. POSTN significantly enhanced the migration of both CRC cells and fibroblasts with FAK, AKT, or STAT3 activation. Co-culture assays demonstrated communication between CRC cells and fibroblasts, enhancing STAT3 and phospho-STAT3 expression in fibroblasts. On the basis of our experiments, we speculated that stromal POSTN accelerated metastasis via enhanced stromal remodeling capacity and activated migration of both tumor and stromal cells. POSTN-modulating therapies may be candidate treatments for patients with CRC.

**Abstract:**

Evidence for the tumor-supporting capacities of cancer-associated fibroblasts (CAFs) has rapidly been accumulating. To uncover clinicopathological importance of periostin (POSTN) expression in colorectal cancer (CRC), the present study immunohistochemically examined its expression status. Furthermore, to reveal its mechanisms involved, molecular experiments were performed. In CRC tissues, 44% of the cases (119/269) exhibited POSTN expression in the CAFs. In contrast, CRC cells expressed POSTN at almost undetectable levels. Survival analyses identified that patients with POSTN-positive CRC had a significantly worse 5-year survival rate (63.2% vs. 81.2%; *p* = 0.011). Univariate analyses revealed that POSTN positivity was associated with peritoneal (*p* = 0.0031) and distant organ metastasis (*p* < 0.001). Furthermore, immunohistochemical analyses identified a significant association between POSTN and p53 complete loss status in CRC cells. Decorin and fibroblast activation protein expression in CAFs was also associated with POSTN. POSTN significantly enhanced the migration of both CRC cells and fibroblasts with FAK and AKT or STAT3 activation, and co-culture assays demonstrated the communication between CRC cells and fibroblasts, which enhanced STAT3 activation in fibroblasts. On the basis of our results, we speculated that stromal POSTN accelerated metastasis via stromal remodeling capacity and activated the migration of both tumor and stromal cells.

## 1. Introduction

Colorectal cancer (CRC) is one of the most common gastrointestinal cancers worldwide, with high morbidity and mortality rates [1]. As for other tumors, evidence for the tumor-supporting capacities of cancer-associated fibroblasts (CAFs) has rapidly been accumulating in CRC. It has been reported that CAFs can modulate cancer cell proliferation, invasion, metastasis, and tumor immunity [2,3,4]. Based on a broad range of tumor-supporting capacities, CAFs are believed to be a promising target of cancer therapy.

Periostin (POSTN), also termed osteoblast-specific factor 2, is a secreted extracellular matrix protein that was originally identified in mesenchymal-lineage cells such as osteoblasts [5]. POSTN plays a critical role in development and tissue regeneration. In knockout mouse models, the absence of *Postn* resulted in growth retardation and dwarfisms, shorter long-bones, and aberrant epiphyseal plate organization [6], suggesting a role for POSTN in bone development, remodeling, and bone strength. Recent studies indicated the contribution of POSTN in dermal regeneration and wound healing, suggesting that POSTN may promote defect closure by facilitating the activation, differentiation, and contraction of fibroblasts [7].

Aberrant POSTN expression with poor clinical outcome has been reported in solid epithelial malignancies including CRC [8,9,10,11]. Recent evidence showed that POSTN, a component of the extracellular matrix (ECM) produced by fibroblasts in the stroma of primary tumors, plays a critical role in the formation and remodeling of cancer tissue microenvironments [8,10,12]. POSTN interacts with cell-surface receptor integrins such as αvβ3, αvβ5, and α6β4 to modulate intracellular signaling pathways in cancer cells and accelerate cell adhesion, survival, invasion, angiogenesis, metastasis, and epithelial–mesenchymal transition (EMT) [8,10,12].

To uncover the clinicopathological importance of periostin expression in colorectal cancer, the present study immunohistochemically examined its expression status. The association with clinicopathological features and impacts on clinical outcome were statistically evaluated. Furthermore, to reveal the mechanisms involved, molecular experiments were performed. Our results suggest the potential utility of POSTN immunohistochemistry in determining the prognosis of CRC. In future, POSTN-modulating therapies may be candidate treatments for patients with CRC.

## 2. Materials and Methods

### 2.1. Tissue Samples

The Institutional Ethical Review Board of Aichi Medical University Hospital approved this project without the requirement to collect patient consent by giving them the opportunity for opt-out. The project was performed in accordance with the Declaration of Helsinki. Two hundred and sixty-nine formalin-fixed, paraffin-embedded (FFPE) samples of primary colorectal tumors resected at Aichi Medical University Hospital from 2009 to 2012 were collected, depending on the availability of tissue samples and clinical information. After surgery, patients were followed up for up to 90 months. All tumors were diagnosed as invasive [13] and naïve to chemotherapy or radiotherapy. Tumors with glandular formation (>50%) or mucus production (>50% of the area) were determined as having a differentiated or mucus-producing histology. A single 4.5 mm core tumor tissue sample derived from an FFPE specimen was assembled into multitumor blocks containing up to 30 samples. All cores were obtained from invasive areas, and approximately 20% of cores contained an invasive front. Non-neoplastic colonic mucosae adjacent to the tumor were also immunohistochemically analyzed.

### 2.2. Immunohistochemistry

The antibodies used in the present study are summarized in Table S1 (Supplementary Materials). Immunohistochemistry was performed using a Leica Bond-Max (Leica Biosystems, Bannockburn, IL, USA) or Ventana BenchMark XT automated immunostainer (Roche Diagnostics, Basel, Switzerland). Signals were visualized using 3,3′-diaminobenzidine. POSTN-, decorin- (DCN), fibroblast activation protein- (FAP), and alpha-smooth muscle actin (α-SMA)-positive areas were evaluated using ImageJ 1.53a software (NIH, Bethesda, MD, USA; Figure S1). p53 immunoreactivity was classified as follows: wild type, overexpression, cytoplasmic, and complete loss [14,15]. The number of phospho-histone H3 (PHH3)-positive cells was counted under the microscope (×400). Cyclin A (CCNA), geminin (GMNN), and Ki-67 labeling indices were determined by counting over 500 tumor cells per case under a high-power field (×400) [16].

### 2.3. Gene Mutation Analyses

*KRAS* mutation status was collected from the medical records. *BRAF* V600E mutation analyses were performed by polymerase chain reaction (PCR)–direct sequencing using the following primers: *BRAF* forward: tgc ttg ctc tga tag gaa aat g; *BRAF* reverse: cag ggc caa aaa ttt aat cag t.

### 2.4. Cells, Plasmids, and Reagents

NIH/3T3 cells were purchased from the American Type Culture Collection (Manassas, VA, USA). CRC cells (Caco-2, COLO205, SW480, CW-2, LoVo, SW48, and HCT-116) were obtained as reported previously [17,18]. Cells were maintained in Dulbecco’s Modified Eagle’s Medium supplemented with 10% fetal bovine serum (FBS).

The lentiviral vectors for full-length human POSTN and control LacZ expressions in NIH/3T3 cells were constructed using the CSII-CMV-MCS-IRES2-Bsd plasmid, which was kindly provided by Dr. H. Miyoshi (RIKEN BioResource Center, Tsukuba, Japan). Recombinant human periostin (rPOSTN, Asn22-Gln836 with a C-terminal 6-His tag) was purchased from R&D systems/Thermo Fisher Scientific (Waltham, MA, USA). Stattic, a selective inhibitor for STAT3, was obtained from FUJIFILM Wako Pure Chemical Corporation (Osaka, Japan).

Immunoblot analyses were performed as previously described [19,20,21]. In brief, whole-cell lysates were prepared using 1× sodium dodecyl sulfate (SDS) sample buffer containing 50 mM Tris-HCl and 2% SDS. Proteins separated by SDS polyacrylamide gel electrophoresis were transferred to a PVDF membrane. Antibody dilutions are summarized in Table S1. Signal intensity was measured by ImageJ software (NIH, Bethesda, MD, USA).

### 2.5. Fluorescence-Activated Cell Sorting (FACS) Analyses

1 × 10^6^ CRC cells were harvested, washed, and stained by using FITC-conjugated antibodies against ITGA6, ITGB4, and their controls on ice for 1 h (Table S1). After staining with 7-AAD (Beckman Coulter, Inc., Brea, CA, USA), the cells were analyzed by using Guava^®^ easyCyte™ systems (Guava Technologies, Inc., Hayward, CA, USA) according to the manufacturer’s protocol. Assays were performed in triplicate.

### 2.6. Cellular Proliferation and Migration Assays

A total of 5 × 10^3^ cells were seeded on 12-well plates. For CRC cells, 500 ng/mL rPOSTN or vehicle was added. After incubation, cell numbers were measured using CellTiter 96 Aqueous One Solution (Promega, Madison, WI, USA) according to the manufacturer’s protocol.

Migration assays were performed using the Falcon Permeable Support for 24-well Plates with 8.0 µm Transparent PET Membrane (Corning, NY, USA) according to the manufacturer’s procedure. Then, 4 × 10^4^ CRC cells or 1 × 10^4^ NIH/3T3 cells per chamber were added to the upper chamber and incubated for 24 h. Ten percent FBS with or without 500 ng/mL rPOSTN was used as a chemoattractant. For the migration assay with co-cultured cells, 1 × 10^5^ NIH/3T3 cells with or without POSTN induction were seeded in the lower chamber. After incubation, cells that had migrated from the upper chamber to the opposite side of the PET membrane were fixed using 100% methanol at room temperature and stained with Giemsa. The number of migrated cells was counted under a microscope.

### 2.7. Co-Culture Assays

To observe the communication between CRC cells and NIH/3T3 cells, the Falcon Permeable Support for 24-well Plates with 0.4 µm Transparent PET Membrane (Corning, NY, USA) was used according to the manufacturer’s procedure. A total of 1 × 10^5^ NIH/3T3 cells with or without POSTN expression was seeded into the lower chamber. After 24 h, 1 × 10^5^ CRC cells were added to the upper chamber. After an additional 24 h incubation, total lysate was extracted and subjected to immunoblot analyses.

### 2.8. Statistical Analyses

Statistical analyses were performed using EZR software version 1.41 [22]. The cutoffs for POSTN immunohistochemistry were defined as the value closest to the upper-left corner in the receiver operating characteristic curves for patient survival at 5 years. The chi-squared test, Fisher’s exact test, Cochran–Armitage trend test, Mann–Whitney U test, or Kruskal–Wallis test was performed to analyze the statistical correlation between categorical data. Simple Bonferroni correction for multiple hypothesis testing was applied for adjustment at a two-sided alpha level of 0.0042 (=0.05/12).

For survival analyses, Kaplan–Meier survival estimates were calculated together with the log-rank test. Survival analyses were also performed using the Kaplan–Meier Plotter program using pan-cancer RNA-seq data according to *POSTN* expression (https://kmplot.com/analysis/, accessed on 1 September 2022). The best cut-off values were automatically set by the program in each tumor type.

## 3. Results

### 3.1. Expression of POSTN in Non-Neoplastic Colonic Mucosa and CRCs

**Figure 1:**
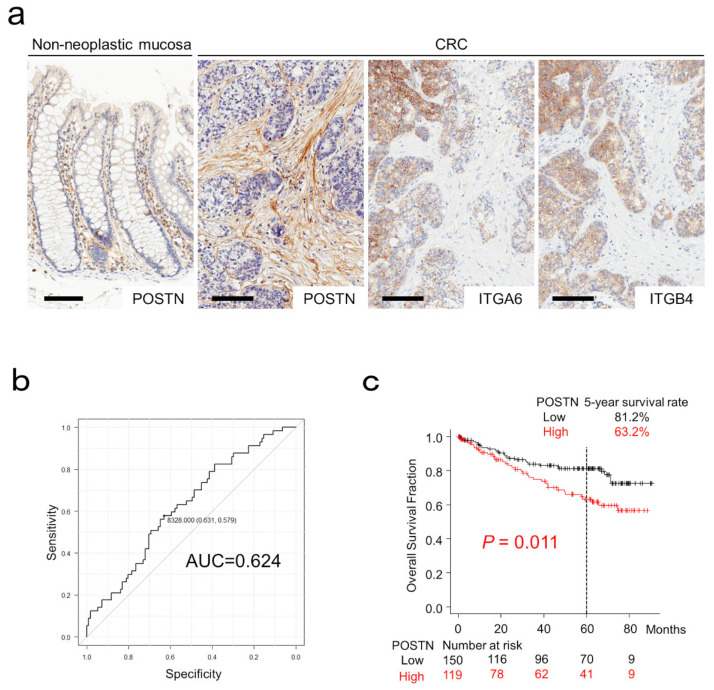
Expression and survival significance of periostin (POSTN) in colorectal cancer (CRC). (**a**) POSTN was weakly expressed on mesenchymal cells of non-neoplastic colonic mucosa. Note that non-neoplastic epithelial cells were negative for POSTN. Cancer-associated fibroblasts, but not CRC cells, expressed POSTN. ITGA6 and ITGB4, the canonical receptors for POSTN, were expressed in CRC cells. Bar, 100 µm. (**b**) ROC curves for POSTN expression on the patient survival at 5 years. (**c**) Kaplan–Meier curves for patients classified by stromal POSTN expression.

Representative images for POSTN, ITGA6, and ITGB4 immunohistochemistry are presented in Figure 1a. In non-neoplastic colonic mucosa, POSTN was weakly expressed in stromal cells within the lamina propria. Non-neoplastic colonic epithelial cells expressed POSTN at almost undetectable levels (Figure 1a). In CRC tissues, 44% of the cases (119/269) exhibited POSTN expression in the CAFs. In addition to non-neoplastic colonic epithelial cells, CRC cells likewise expressed POSTN at almost undetectable levels (Figure 1a). ITGA6 and ITGB4, which are considered to be canonical receptors for POSTN, were expressed in CRC cells adjacent to the POSTN-positive CAFs (Figure 1a).

### 3.2. Survival Analyses of Patients with CRC and Other Tumors

The cut-off value for POSTN expression was set at 8328 pixels according to patient death at 5 years (Figure 1b). Patients with POSTN-positive CRC had a significantly worse 5-year survival rate (63.2% vs. 81.2%; *p* = 0.011; Figure 1c).

Among the analyzed tumors using Kaplan–Meier Plotter pan-cancer RNA-seq data, many tumor types, including stomach and pancreatic cancers, showed significantly higher risk in POSTN-expressing cases (HR = 1.37–2.43; Table S2).

### 3.3. Characteristics of CRCs Classified According to POSTN Expression

The clinical, pathological, and immunohistochemical features of the analyzed tumors are summarized in Table 1 according to stromal POSTN expression. POSTN positivity was significantly associated with peritoneal metastasis (*p* = 0.0031) and distant organ metastasis (*p* < 0.001), and tended to be associated with pT stage (*p* = 0.0098) and incomplete resection status (*p* = 0.043; Table 1).

**Figure 2:**
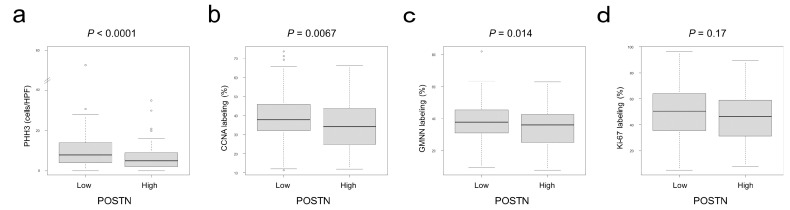
Correlation between stromal POSTN expression and cellular proliferation markers in CRC cells. Cellular proliferation marker expression in CRC cells classified by stromal POSTN expression. (**a**) PHH3, (**b**) CCNA, (**c**) GMNN, and (**d**) Ki-67. POSTN-positive tumors contained a significantly lower number of PHH3-, CCNA-, and GMNN-positive cells than negative tumors. The circles indicate outliers.

In the association with cellular proliferation markers, POSTN showed significantly inverse associations with PHH3, CCNA, and GMNN. No significant association was detected between POSTN and Ki-67 labeling index (Figure 2).

**Figure 3:**
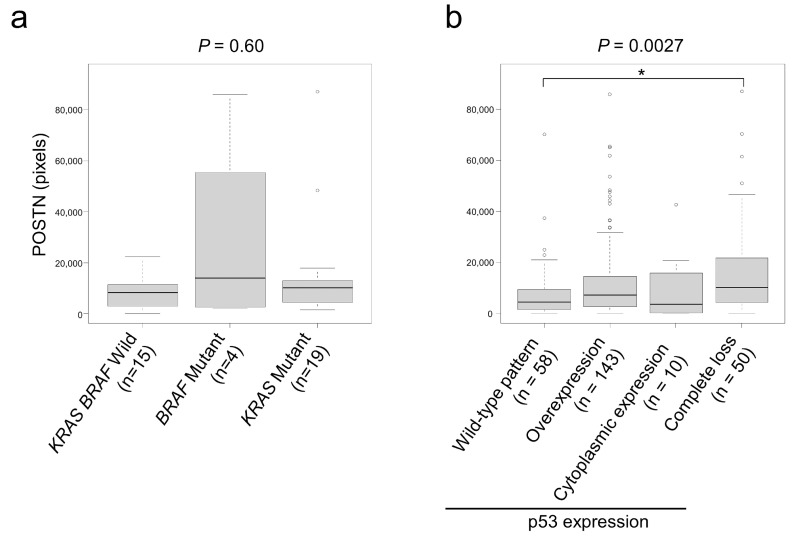
Association between gene mutation status and stromal POSTN expression. (**a**) Association between *KRAS*/*BRAF* mutation and stromal POSTN expression. (**b**) Associations between p53 expression patterns and POSTN expression. CRCs showing p53 complete loss expressed significantly higher levels of stromal POSTN than p53 wild-type tumors. The circles indicate outliers. *, *p* < 0.05.

Regarding *KRAS* and *BRAF* mutation status, *BRAF* mutants tended to show higher POSTN expression (Figure 3a). Based on the usefulness of p53 immunohistochemistry as a surrogate marker to predict *TP53* mutation status, we classified our cohort according to p53 expression status as follows: wild type, overexpression, cytoplasmic, and complete loss. Among them, patients with p53 complete loss tumors showed significantly higher POSTN expression than those with p53 wild-type pattern (Figure 3b).

### 3.4. Characteristics of POSTN-Expressing CAFs in CRC

**Figure 4:**
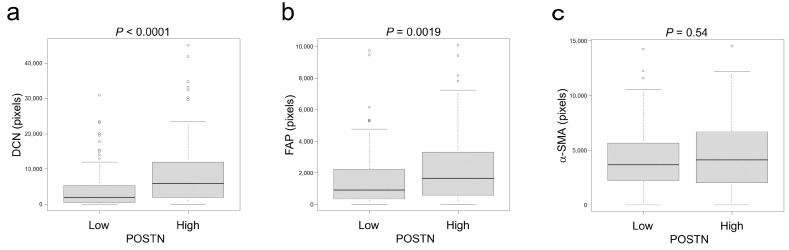
Association between stromal POSTN and CAF marker expression. (**a**–**c**) Association between POSTN and CAF marker expression. (**a**) DCN, (**b**) FAP, and (**c**) α-SMA. POSTN was significantly associated with DCN and FAP but not α-SMA. The circles indicate outliers.

CAFs are heterogeneous and present various gene expression patterns. Recent studies identified several markers specifically expressed in CAFs [23]. In the present study, to characterize POSTN-positive CAFs, the association between POSTN and α-SMA, FAP, or DCN was analyzed. Among these, DCN and FAP were significantly associated with POSTN expression. In contrast, no significant association was identified between α-SMA and POSTN (Figure 4).

### 3.5. POSTN Expression in Cultured CRC Cells

**Figure 5:**
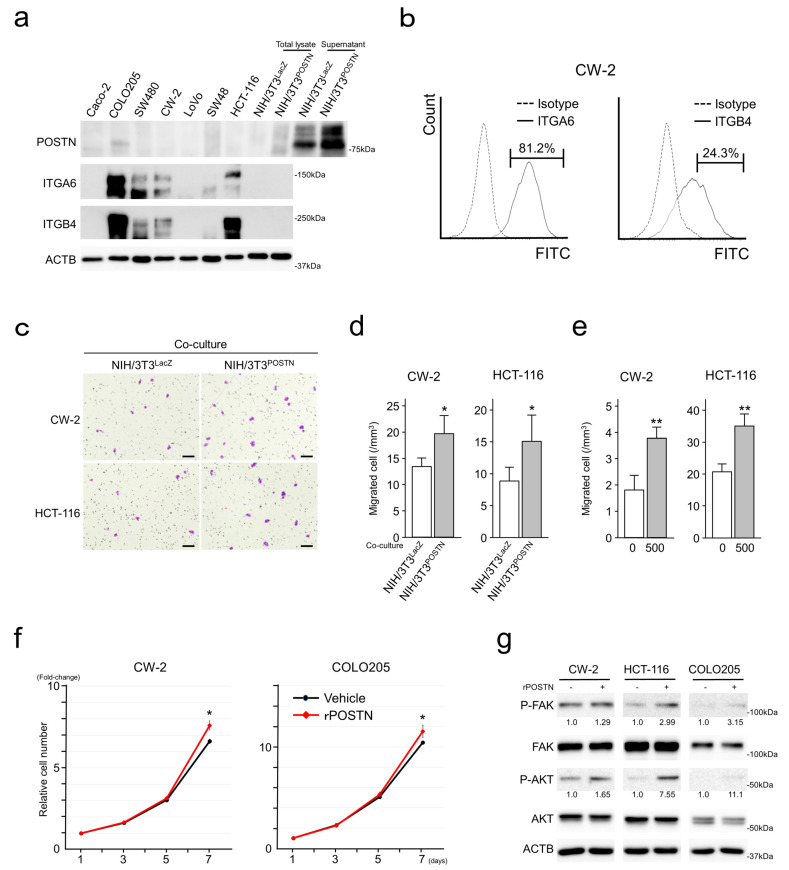
Effects of POSTN on CRC cells. (**a**) Immunoblot analysis showing POSTN and ITGs in CRC and NIH/3T3 cells. (**b**) Results for FACS analyses showing ITGA6 and ITGB4 expression on CW-2 cells. (**c**–**e**) Results of migration assays showing that co-culture with NIH/3T3^POSTN^ or addition of rPOSTN in the lower chamber accelerated the migration of CRC cells. Bar = 100 µm. Assays were performed in quadruplicate. Data are shown as mean ± S.D. *, *p* < 0.05, **, *p* < 0.01. (**f**) Addition of rPOSTN to the culture medium enhanced the proliferation of CRC cells. Assays were performed in triplicate. Data are shown as mean ± S.D. *, *p* < 0.05. (**g**) Immunoblot analysis showing upregulated P-FAK and P-AKT in rPOSTN-stimulated CRC cells. The uncropped bolts are shown in Supplementary Material File S1.

In cultured CRC cells, similar to the results from the immunohistochemistry for CRC tissues and past reports [10], POSTN was expressed at almost undetectable levels. NIH/3T3^POSTN^ secreted high levels of POSTN into the culture medium. CRC cells variably expressed ITGA6 and ITGB4 (Figure 5a). FACS analyses identified ITGA6 and ITGB4 expressions on the surface of CRC cells (Figure 5b and Figure S2). CRC cells expressed ITGAV, ITGA4, ITGA5, ITGB1, ITGB3, and ITGB5 at almost undetectable levels.

### 3.6. POSTN Enhanced Migration and Proliferation of CRC Cells

To confirm the effects of POSTN on CRC cells, we first performed Transwell migration assays. Co-culture of NIH/3T3^POSTN^, secreting higher levels of POSTN, in the lower chamber significantly enhanced the migration of CW-2 and HCT-116 cells (Figure 5c,d), indicating that secreted POSTN from NIH/3T3^POSTN^ served as a chemoattractant for CRC cells. To confirm this notion, recombinant POSTN (rPOSTN) was added to the culture medium in the lower chamber along with 10% FBS. As expected, rPOSTN significantly enhanced the migration of CRC cells (Figure 5e).

Along with the enhanced migration, CRC cell proliferation was slightly but significantly accelerated by rPOSTN with the upregulation of phosphorylated FAK and AKT (Figure 5f,g).

### 3.7. POSTN Accelerated Migration but Suppressed Proliferation of Fibroblasts

**Figure 6:**
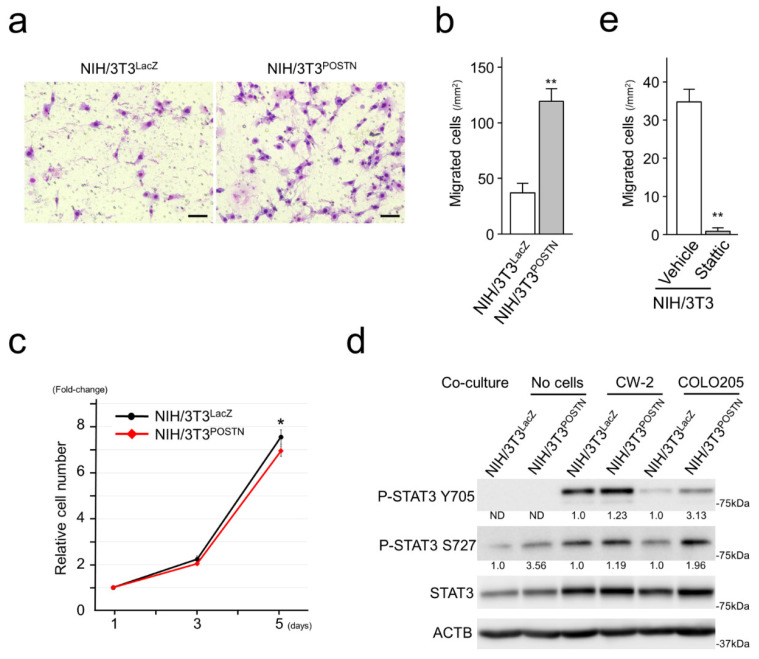
POSTN enhanced the motility but suppressed the proliferation of NIH/3T3 cells. (**a**,**b**) Exogenous POSTN enhanced the migration of NIH/3T3 cells. Bar = 100 µm. Assays were performed in quadruplicate. Data are shown as mean ± S.D. **, *p* < 0.01. (**c**) POSTN suppressed the cellular proliferation of NIH/3T3 cells. Assays were performed in triplicate. Data are shown as mean ± S.D. *, *p* < 0.05. (**d**) Immunoblot analysis showing upregulated STAT3 and P-STAT3 in NIH/3T3 cells. Exogeneous POSTN upregulated P-STAT3 at S727. Co-culture with CRC cells upregulated basal STAT3 and P-STAT3 at Y705 in NIH/3T3. (**e**) Stattic, a selective inhibitor for STAT3, significantly downregulated the migration of NIH/3T3 cells at 10 µM. Assays were performed in quadruplicate. Data are shown as mean ± S.D. **, *p* < 0.01. The uncropped bolts are shown in Supplementary Material File S1.

Forced POSTN expression in NIH/3T3 cells significantly enhanced cell motility with upregulation of phosphorylated STAT3 at serine 727 (S727; Figure 6a,b,d). Regarding cellular proliferation, POSTN slightly suppressed the proliferation of NIH/3T3 cells (Figure 6c).

It was reported that cancer cells and fibroblasts communicate with each other. To reveal the communication between CRC cells and fibroblasts *in vitro*, we performed co-culture assays. Co-culture with CRC cells upregulated the basal expression levels of STAT3 in NIH/3T3 cells. Furthermore, upregulation of phosphorylated STAT3 at tyrosine 705 (Y705) was observed in NIH/3T3 cells co-cultured with CRC cells (Figure 6d). Note that NIH/3T3^POSTN^ expressed higher levels of phosphorylated STAT3 at Y705 than NIH/3T3^LacZ^. Stattic, a selective STAT3 inhibitor significantly suppressed the migration of NIH/3T3 (Figure 6e).

## 4. Discussion

CAFs are a major component of the cancer stroma and are a highly heterogeneous population of cells with different functions probably resulting from their different origins: resident fibroblasts [24,25], bone-marrow-derived mesenchymal stromal cells [26], mature adipocytes [27], and other cells exist within tumor microenvironments [4]. Another source of CAFs could even be tumor cells after the process of EMT [4]. Based on the heterogeneity of CAFs, specific and common markers for CAF have not been identified; however, many attempts have been made to identify markers that can classify CAFs. In CRC, CAFs have been classified into two types, CAF-A and CAF-B, according to their gene expression status, detected by single-cell sequencing [23]. CAF-B has been reported to express cytoskeletal genes and other known markers of activated myofibroblasts. In contrast, CAF-A has been defined by its expression of DCN, FAP, MMP2, and COL1A2, indicating its extracellular matrix-remodeling capacity [23]. In the present study, POSTN expression was significantly associated with DCN and FAP expression but not with α-SMA (Figure 4). POSTN-positive CAFs may harbor gene expression statuses and/or phenotypes similar to CAF-A.

The impact of CAFs on cancer invasion and metastasis occurs through remodeling of the ECM, modulation of EMT in cancer cells, and secretion of growth factors supporting cancer cells [4,28,29]. CAFs promote ECM remodeling by generating ECM tracks, secreting factors (enzymes, miRNAs, and exosomes), and inducing matrix stiffness [30]. During tumor progression, CAFs can generate ECM tracks to modify the ECM, making it more permissive for tumor invasion into the surrounding tissue [30]. In the present study, POSTN positivity was significantly associated with peritoneal and distant organ metastasis (Table 1). Furthermore, it tended to associate with pT stage and incomplete resection status (Table 1). These observations may be due to the POSTN-positive CAFs, the CAF-A-like extracellular matrix-remodeling capacity, and the enhanced motility of both cancer cells and fibroblasts (Figure 5 and Figure 6).

Cancer cells and CAFs communicate with each other. Aberrantly expressed POSTN by stromal cells modulates intracellular signaling pathways of cancer cells and accelerates many types of malignant phenotypes including migration, invasion, and EMT [8,10,11,12]. At the same time, stromal POSTN has been reported to enhance IL-6 production in CRC cells and create a positive feedback loop between fibroblasts and CRC cells to promote CRC development by canonical IL-6/JAK/STAT3 activation in CAFs [8,31]. In the present study, POSTN accelerated CRC cell proliferation and migration with activation of FAK and AKT signaling (Figure 5). Furthermore, in vitro co-culture experiments identified the upregulation of basal STAT3 and tyrosine 705 (Y705)-phosphorylated STAT3 in fibroblasts co-cultured with CRC cells (Figure 6), indicating activation of the canonical IL-6/JAK/STAT3 pathway due to the CRC cells. The canonical IL-6/JAK/STAT3 pathway accelerates STAT3 dimerization and nuclear translocation, allowing STAT3 to act as a transcription factor [32]. Interestingly, forced expression of POSTN itself upregulated serine 727 (S727) phosphorylation of STAT3 in fibroblasts. The mechanisms triggering this modification and the function of phosphorylated S727 are still debated [32]. Several kinases, such as extracellular signal-regulated kinase (ERK) 1, ERK2, mitogen-activated protein kinase (MAPK) p38, and c-Jun N-terminal kinase (JNK)**,** are thought to be responsible for this modification [33]. S727 phosphorylation has often been considered to be an enhancer of STAT3 nuclear transcriptional activity that probably acts by recruiting activating cofactors [33]. Inhibition of POSTN may be a promising therapeutic strategy against solid malignancies via repression of both cancer and stromal cells.

CRC cell proliferation was slightly accelerated by rPOSTN in vitro (Figure 5). In contrast, histological analyses identified an inverse correlation between stromal POSTN and cell proliferation marker expression in CRC cells (Figure 2). It was reported that CRC cell proliferation decreases according to the pT stage [16,34]. In the present study, even in the pT stage-matched analyses, stromal POSTN tended to be inversely associated with cell proliferation marker expression in CRC cells. Based on our observations, the authors consider that factor(s) other than POSTN in stromal cells dominantly regulate the proliferation of CRC cells in vivo.

POSTN expression in CAFs has been reported to be weakly associated with *BRAF* mutations (*p* = 0.046) in CRCs [9]. In the present study, cases carrying *BRAF* mutations tended to show higher POSTN expression than *KRAS*/*BRAF* wild and *KRAS* mutant cases (Figure 3a); however, a significant association was not identified. In contrast, stromal POSTN expression in tumors with complete loss of p53 was significantly higher than in other tumors (Figure 3b). In our cohort, among the p53 expression patterns, complete loss of p53 expression uniquely showed a worse clinical outcome [14]. We are intrigued by these observations because of the possibility that *TP53* gene mutation status in CRC cells may affect the gene expression status of CAFs, indicating a close association between CRC cells and CAFs. p53 is a transcription factor, and mutations in *TP53* result in different isoforms with variable transcriptional activity, which leads to different cancer phenotypes [35]. The disruption of the transcriptional activity of p53 by its mutation is complicated; the transcriptional activity of p53 was significantly affected by the mutation types and/or its position. Co-culture experiments of fibroblasts with CRC cells with induced *TP53*-knockout by genome editing may reveal the mechanism(s) behind our observations.

The limitations of this study include the number of CRC patients, especially those with *BRAF* mutations. A larger cohort with gene mutation and comorbidity information may be needed to identify the additional clinical significance of POSTN expression in CRC. Another limitation was the unavailability of cultured fibroblasts of human non-neoplastic and/or neoplastic colorectal origin. In the present study, NIH/3T3 cells were used as a model. The use of fibroblasts and/or CAFs of human colorectal origin may identify additional molecular signaling pathways between CRC cells and fibroblasts or CAFs.

## 5. Conclusions

The present study identified worse clinical outcomes in patients with CRC expressing stromal POSTN. Significant associations between stromal POSTN expression and tumor metastasis to the peritoneum and distant organs were also identified. Based on our results, we speculated that stromal POSTN accelerated the metastasis of CRC cells via a CAF-A-like ECM-remodeling capacity and activated the migration of both tumor and stromal cells. Immunohistochemistry for POSTN could be used for the prognostication of patients with CRC, while POSTN-modulating therapies may be candidate treatments for patients with CRC.

## Figures and Tables

**Table 1:** Characteristics of colorectal cancers (CRCs) classified by stromal periostin (POSTN) expression.

			POSTN		
		Total No.	Positive	Negative	*p*-Value
		269 (100%)	119 (44%)	150 (56%)	
Sex					
	Male	143 [53%]	62 [52%]	81 [54%]	0.85 ^a^
	Female	126 [47%]	57 [48%]	69 [46%]	
Age, years (mean ± S.D.)		68.6 ± 12.6	69.40 ± 12.55	67.99 ± 12.67	0.36 ^b^
Size, cm (mean ± S.D.)		5.0 ± 2.6	5.22 ± 2.26	4.82 ± 2.66	0.21 ^b^
Tumor location					
	Right-sided colon	124 [46%]	56 [47%]	68 [45%]	0.18 ^a^
	Left-sided colon	86 [32%]	32 [27%]	54 [36%]	
	Rectum	59 [22%]	31 [26%]	28 [19%]	
pT stage					
	pT2	36 [13%]	8 [7%]	28 [19%]	0.0098 ^c^
	pT3	189 [70%]	88 [74%]	101 [67%]	
	pT4	44 [16%]	23 [19%]	21 [14%]	
Histological differentiation					
	Well to moderately	242 [90%]	105 [88%]	137 [91%]	0.53 ^a^
	Poorly	27 [10%]	14 [12%]	13 [9%]	
Mucus production					
	Positive	14 [5%]	8 [9%]	6 [3%]	0.47 ^a^
	Negative	255 [95%]	111 [91%]	144 [97%]	
Lymph node metastasis					
	Positive	124 [49%]	59 [52%]	65 [46%]	0.43 ^a^
	Negative	129 [51%]	54 [48%]	75 [54%]	
Peritoneal metastasis					
	Positive	50 [19%]	32 [27%]	18 [12%]	0.0031 ^a^
	Negative	219 [81%]	87 [73%]	132 [88%]	
Distant organ metastasis					
	Positive	44 [16%]	30 [25%]	14 [9%]	<0.001 ^a^
	Negative	225 [84%]	89 [75%]	136 [91%]	
Operation status					
	Complete resection	237 [88%]	99 [83%]	138 [92%]	0.043 ^a^
	Incomplete resection	32 [12%]	20 [17%]	12 [8%]	
MMR system status					
	Deficient	31 [12%]	13 [11%]	18 [12%]	0.94 ^a^
	Preserved	238 [88%]	106 [89%]	132 [88%]	

^a^*p*-values were calculated by the chi-squared test for POSTN expression. ^b^*t*-test or ^c^ Cochran–Armitage trend test was used to calculate *p*-values. The Bonferroni-corrected *p*-value for significance was *p* = 0.0042 (0.05/12).

## Data Availability

The datasets used and/or analyzed during the present study are available from the corresponding author on reasonable request.

## Supplementary Materials

The following supporting information can be downloaded at https://www.mdpi.com/article/10.3390/cancers15030606/s1. Figure S1: Representative images for the measurement of POSTN expression; Figure S2: FACS analyses of CRC cells; Table S1: Antibodies and Reagents for Immunohistochemistry, Immunoblotting and FACS analysis; Table S2: Survival analyses in Kaplan-Meier Plotter according to POSTN expression. Representative images for the measurement of POSTN-positive areas. File S1: Original blots of Figure 1a, Figure 5a,c,g, and Figure 6a,d.
