# Supplementary material for: Stromal POSTN Enhances Motility of Both Cancer and Stromal Cells and Predicts Poor Survival in Colorectal Cancer
Source: Cancers (Basel). 2023 Jan 18;15(3):606. doi: 10.3390/cancers15030606 (PMC9913098; doi:10.3390/cancers15030606)

Table S1. Antibodies and Reagents for Immunohistochemistry, Immunoblotting and FACS analysis

	IHC		IB	FACS	
Proteins	Reagent	Dilution	Dilution	Dilution	Antibodies
ACTB	-	-	5,000	-	AC-74, SIGMA (Rocklin, CA)
AKT	-	-	1,000	-	#9272, Cell signaling Technology, Inc. (Danvers, MA)
P-AKT (Ser 473)	-	-	1,000	-	Clone D9E, Cell signaling Technology, Inc. (Danvers, MA)
CCNA	IV	100	-	-	sc-751, Santa Cruz Biothechnology, Inc. (Dallas, TX)
Decorin	OV	200	-	-	ab268048, Abcam (Cambridge, UK)
FAK	-	-	1,000	-	Clone D2R2E, Cell Signaling Technology, Inc. (Danvers, MA)
P-FAK (Tyr 397)	-	-	1,000	-	Clone D20B1, Cell Signaling Technology, Inc. (Danvers, MA)
FAP	BM	200	-	-	EPR20021, Abcam (Cambridge, UK)
Geminin	OV	100	-	-	EPR14637, Abcam (Cambridge, UK)
ITGAV	-	-	1,000	-	#4711, Cell Signaling Technology, Inc. (Danvers, MA)
ITGA4	-	-	1,000	-	Clone D2E1, Cell Signaling Technology, Inc. (Danvers, MA)
ITGA5	-	-	1,000	-	#4705, Cell Signaling Technology, Inc. (Danvers, MA)
ITGA6	-	-	1,000	-	#3750, Cell Signaling Technology, Inc. (Danvers, MA)
ITGA6	BM	500	-	-	EPR18124, Abcam (Cambridge, UK)
ITGA6	-	-	-	20	FITC conjugated, #313605, BioLegend (San Diego, CA)
ITGB1	-	-	1,000	-	Clone D2E5, Cell Signaling Technology, Inc. (Danvers, MA)
ITGB4	BM	200	1,000	-	Clone D8P6C, Cell Signaling Technology, Inc. (Danvers, MA)
ITGB4	-	-	-	20	FITC conjugated, #327805, BioLegend (San Diego, CA)
Ki-67	OV	100	-	-	Clone MIB-1, Dako/Agilent (Santa Clara, CA)
MLH1	OV	200	-	-	Clone G168-728, BD Biosciences (Franklin Lakes, NJ)
Mouse IgG2a	-	-	-	20	FITC conjugated, #400209, BioLegend (San Diego, CA)
MSH2	OV	200	-	-	Clone G219-1129, BD Biosciences (Franklin Lakes, NJ)

MSH6	OV	400	-	-	Clone 44/MSH6, BD Biosciences (Franklin Lakes, NJ)
Periostin	BM	200	1,000	-	Ab92460, Abcam (Cambridge, UK)
PMS2	OV+Linker	50	-	-	Clone A16-4, BD Biosciences (Franklin Lakes, NJ)
PHH3	OV	500	-	-	Cell Marque™, Millipore SIGMA (Rocklin, CA)
p53	BM	100	-	-	Clone DO7, Leica Biosystems (Wetzlar, Germany)
α-SMA	BM	600	-	-	Clone 1A4, Dako/Agilent (Santa Clara, CA)
Stat3 α	-	-	1,000	-	#8768, Cell Signaling Technology, Inc. (Danvers, MA)
P-Stat3 (Tyr 705)	-	-	1,000	-	Clone D3A7, Cell Signaling Technology, Inc. (Danvers, MA)
P-Stat3 (Ser 727)	-	-	1,000	-	#9134S, Cell Signaling Technology, Inc. (Danvers, MA)
Rat IgG2a	-	-	-	20	FITC conjugated, #400505, BioLegend (San Diego, CA)

IHC, immunohistochemistry. IB, immunoblotting. FACS, fluorescent-activated cell sorting. IV, iView reagent. OV, OptiView reagent. BM, BondMax.

Table S2. Survival analyses in Kaplan-Meier Plotter according to *POSTN* expression

	Patient No.	Hazard Ratio	95% CI min	95% CI max	Log-rank *P*-value
Kidney papillary renal cell carcinoma	287	2.43	1.34	4.39	0.0025
Esophageal adenocarcinoma	80	2.25	1.02	4.96	0.039
Cervical squamous carcinoma	304	2.20	1.34	3.62	0.0014
Stomach adenocarcinoma	371	1.83	1.31	2.57	0.00031
Pancreatic ductal adenocarcinoma	177	1.69	1.03	2.78	0.038
Sarcoma	259	1.66	1.11	2.47	0.012
Hepatocellular carcinoma	370	1.66	1.17	2.36	0.0042
Uterine endometrial carcinoma	542	1.63	1.05	2.53	0.027
Bladder carcinoma	404	1.57	1.12	2.20	0.0087
Kidney clear cell renal cell carcinoma	530	1.55	1.12	2.14	0.007
Lung adenocarcinoma	504	1.47	1.08	1.98	0.013
Lung squamous cell carcinoma	495	1.48	1.13	1.94	0.0041
Head-neck squamous cell carcinoma	499	1.37	1.04	1.79	0.022

RNA-seq data were analyzed using the Kaplan–Meier Plotter program on the web site. Note that no significant difference was detected in breast cancer, esophageal squamous cell carcinoma, ovarian cancer, pheochromocytoma and paraganglioma, rectal adenocarcinoma, testicular germ cell tumor, thymoma, and thyroid carcinoma.

Figure S1. Representative images for the measurement of POSTN expression

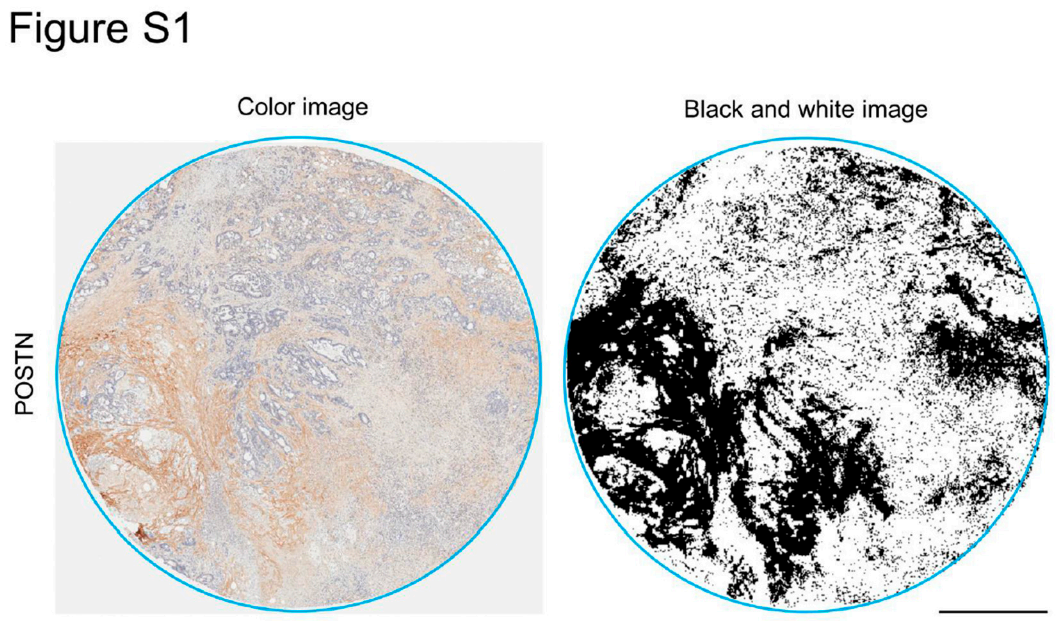

Figure S2. FACS analyses of CRC cells.

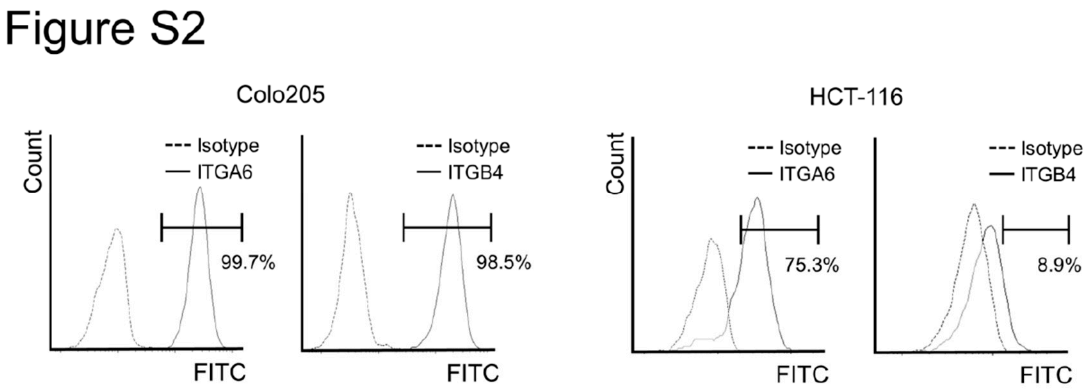

Supplement: Supplementary file 1 [file cancers-15-00606-s001.zip › cancers-2075681-supplementary.pdf]
