# Supplementary figures and images for: Stromal POSTN Enhances Motility of Both Cancer and Stromal Cells and Predicts Poor Survival in Colorectal Cancer
Source: Cancers (Basel). 2023 Jan 18;15(3):606. doi: 10.3390/cancers15030606 (PMC9913098; doi:10.3390/cancers15030606)

Figure 1a

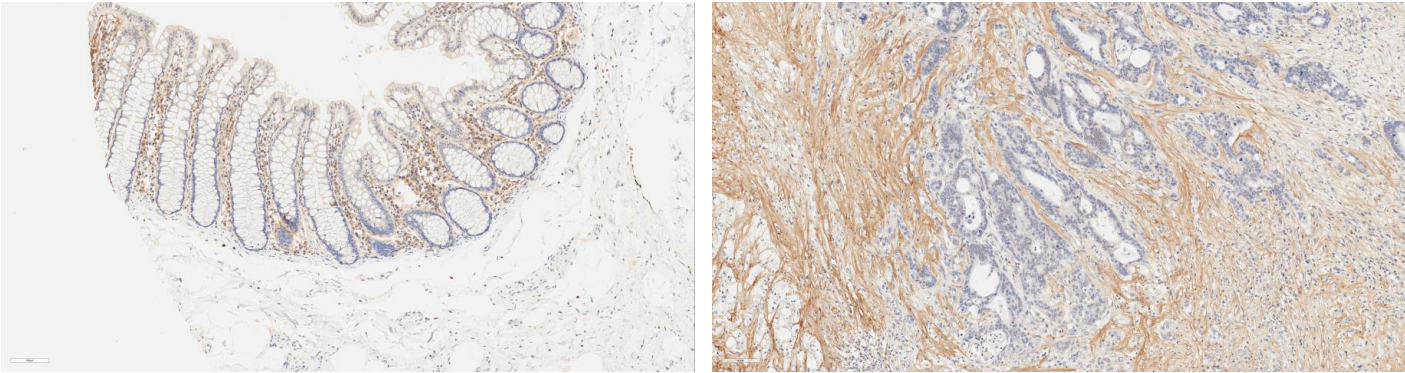

Figure 5a

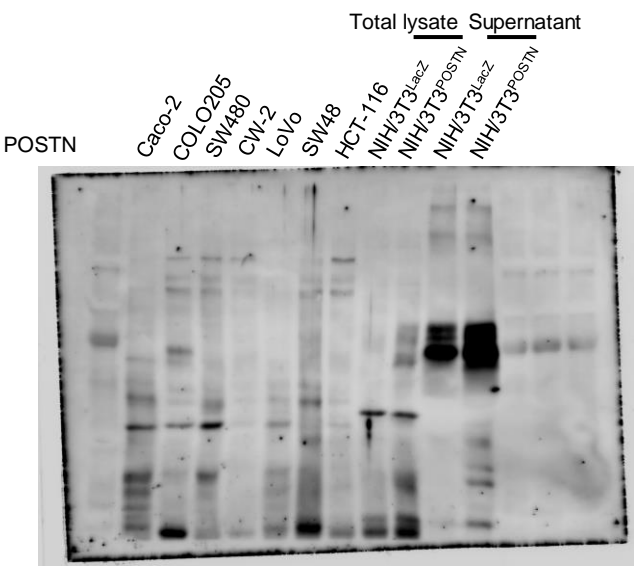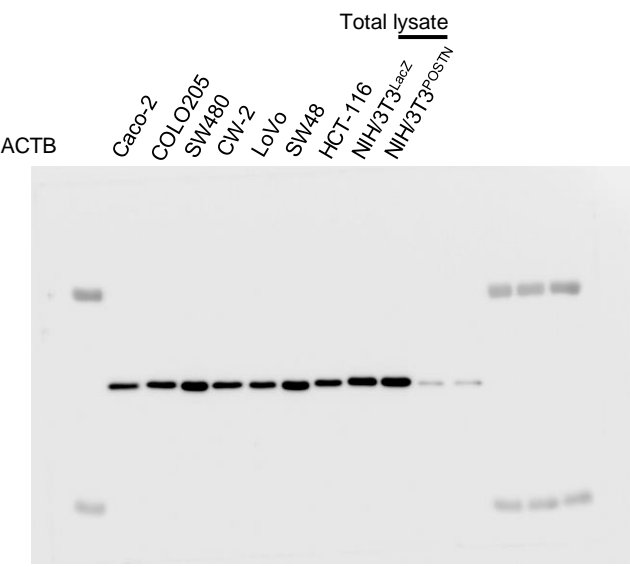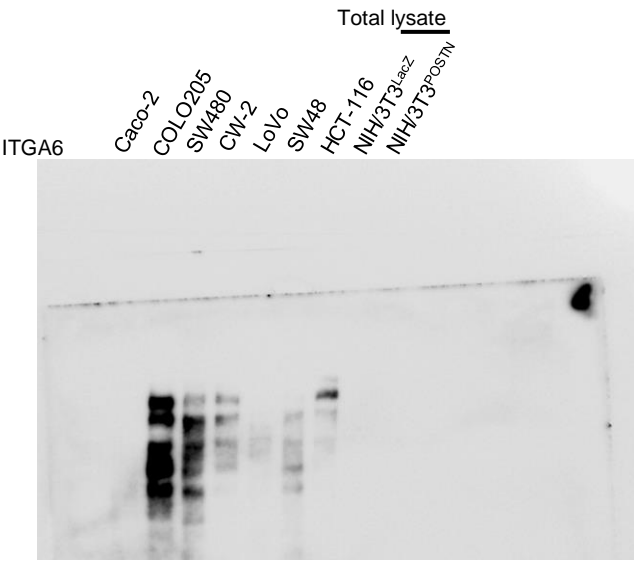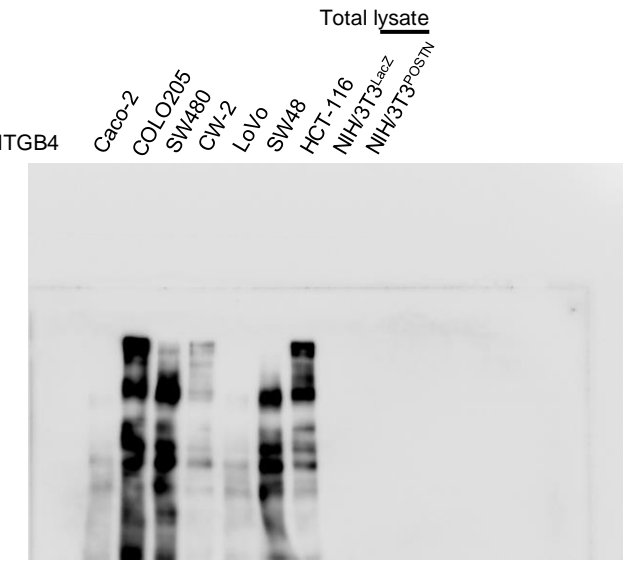

Figure 5c

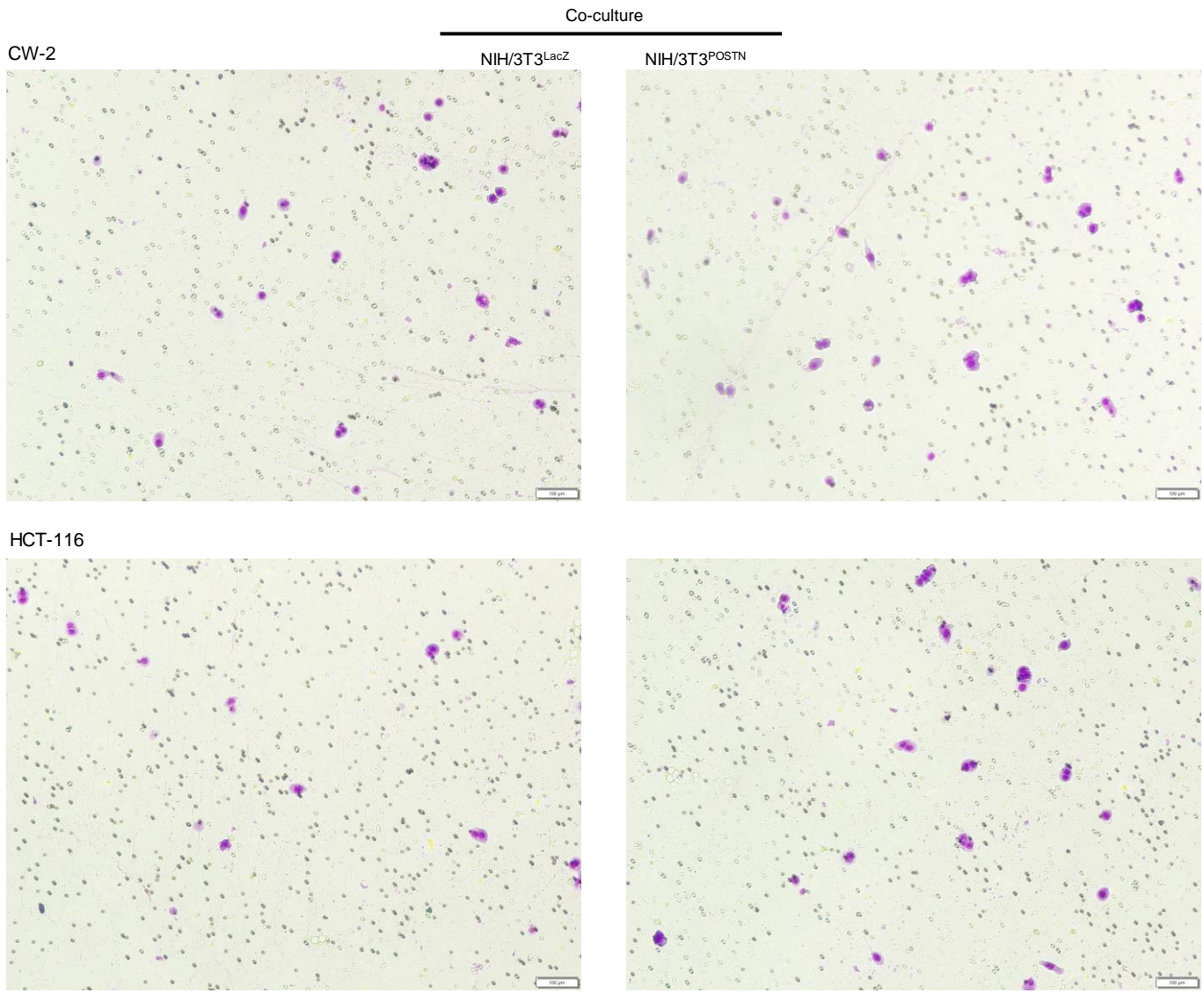

Figure 5g

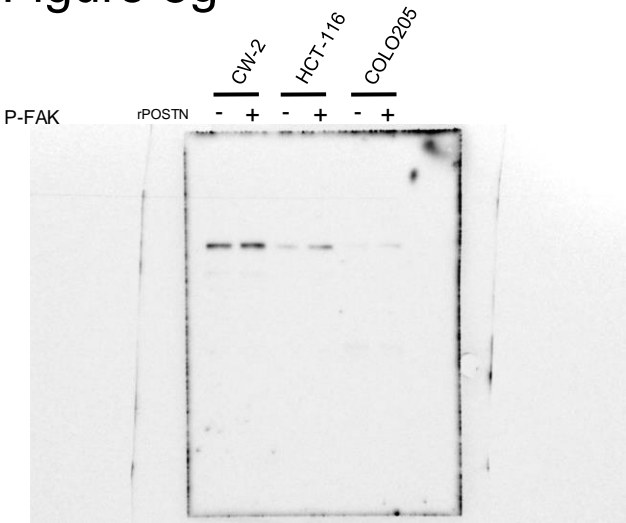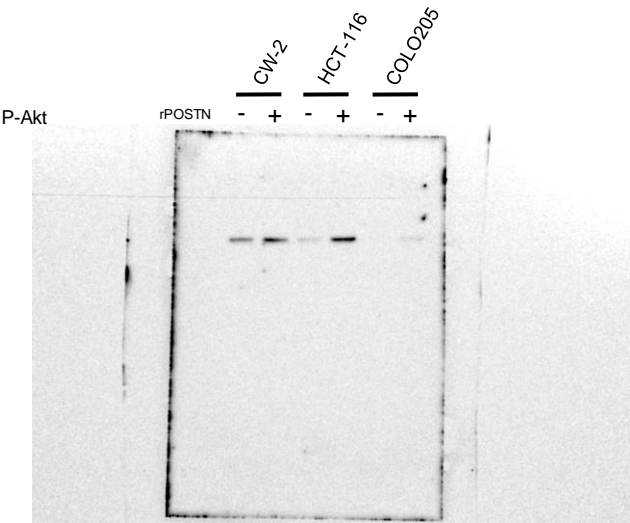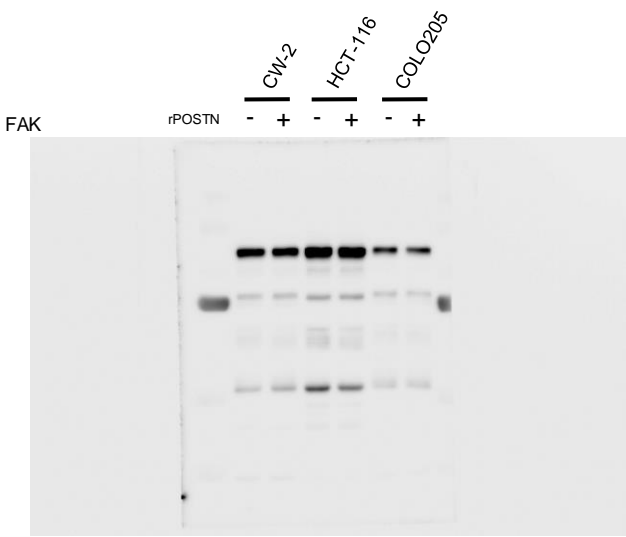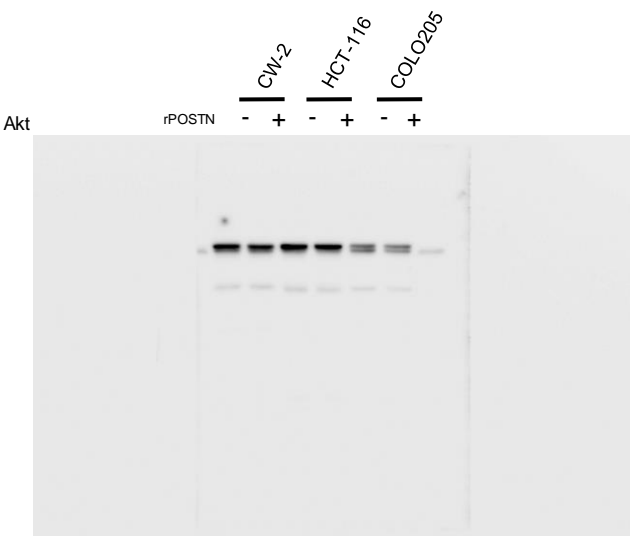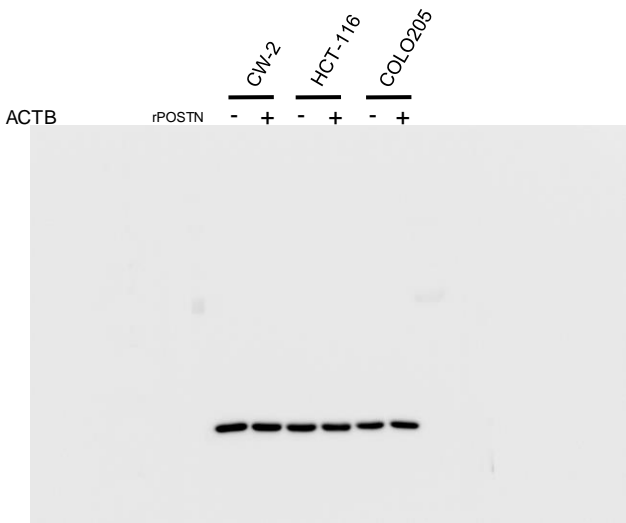

Figure 6a

NIH/3T3<sup>LacZ</sup>

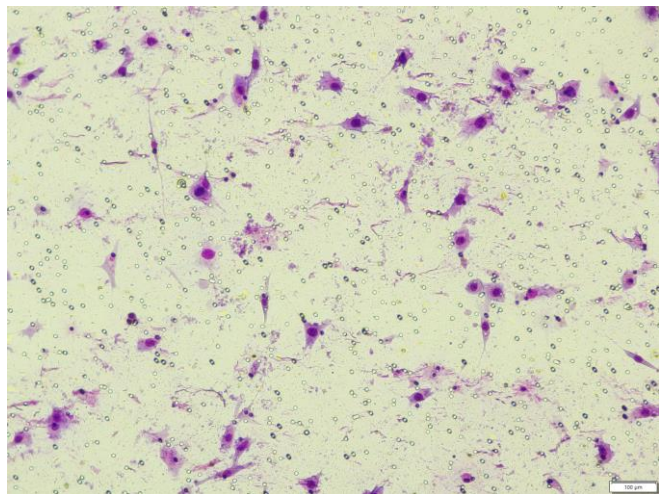

NIH/3T3<sup>POSTN</sup>

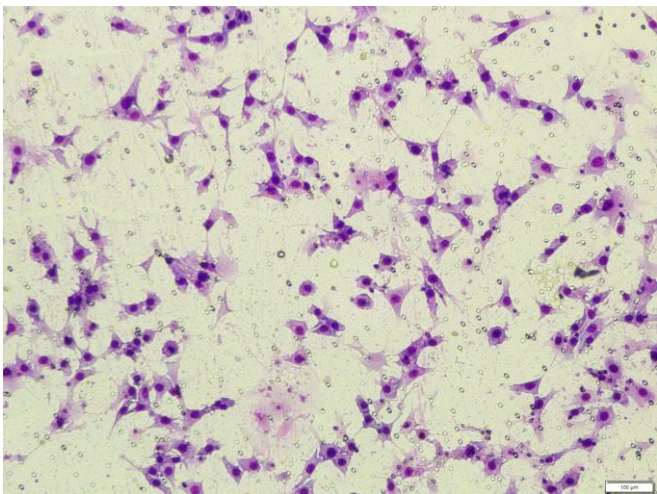

Figure 6d

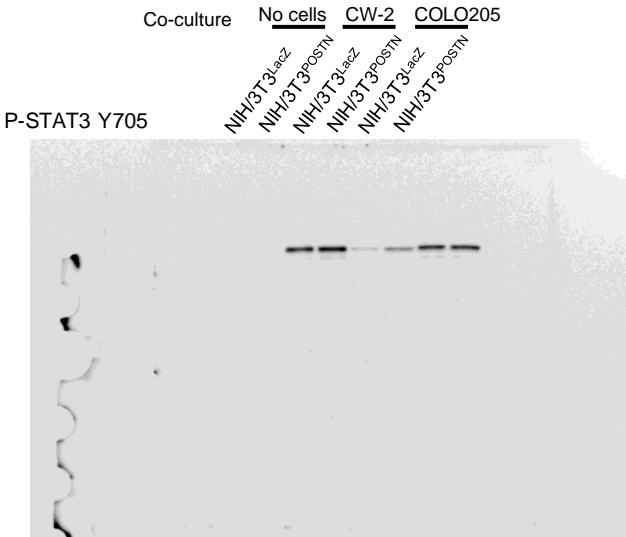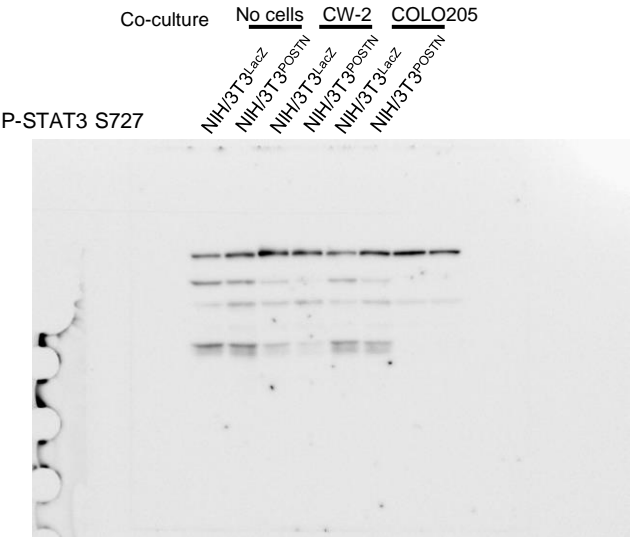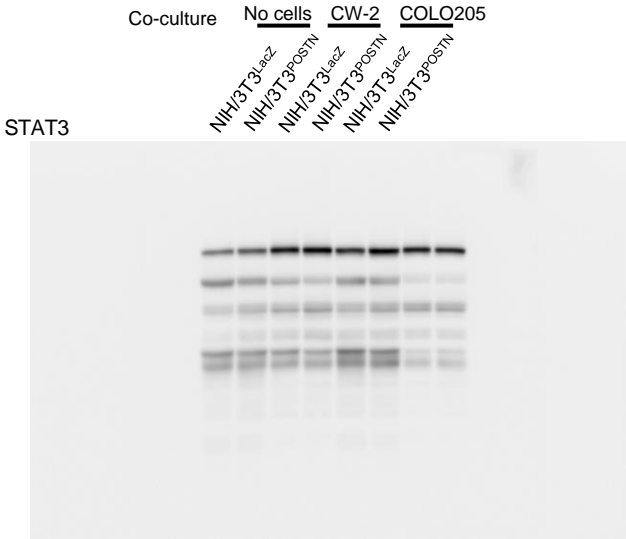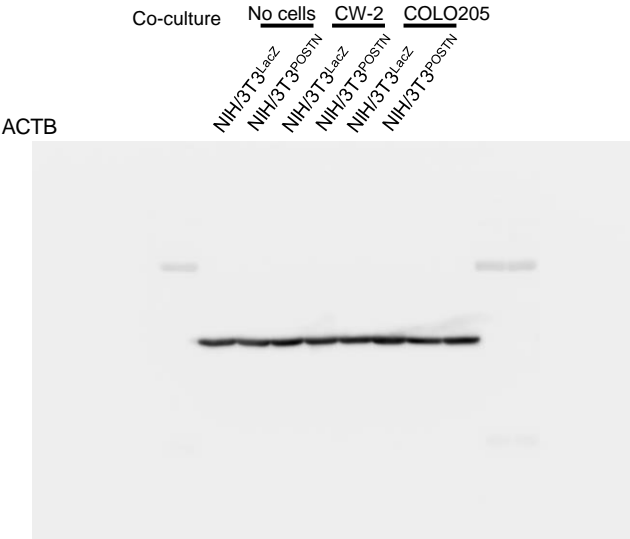

Supplement: Supplementary file 1 [file cancers-15-00606-s001.zip › cancers-2075681-File S1-original-images.pdf]
